# Unusual multifocal intraosseous papillary intralymphatic angioendothelioma (Dabska tumor) of facial bones: a case report and review of literature

**DOI:** 10.1186/1746-1596-8-160

**Published:** 2013-09-24

**Authors:** Bin LI, Yang Li, Xiao-ying Tian, Zhi Li

**Affiliations:** 1 Department of Pathology, The First Affiliated Hospital, Sun Yat-sen university, 58, Zhongshan Road II, Guangzhou 510080, China; 2 School of Chinese Medicine, Hong Kong Baptist University, 7, Baptist University Road, Kowloon Tong, Hong Kong, China

**Keywords:** Papillary intralymphatic angioendothelioma, Dabska tumor, Osteolytic lesion, Differential diagnosis

## Abstract

**Abstract:**

Papillary intralymphatic angioendothelioma (PILA) or Dabska tumor is extremely rare, and often affects the skin and subcutaneous tissues of children. Since its first description by Dabska, only a few intraosseous cases have been described in the literature and none of them presents with multifocal osteolytic lesion of bones. We present a case of unusual multifocal intraosseous PILA in facial bones occurring in a 1 year 3 month old male child. Computed tomography (CT) scan revealed multifocal osteolytic lesions were located at the left zygoma, left orbital bone and right maxillary. Histologically, the lesions were ill-defined and composed of multiple delicate interconnecting vascular channels with papillae formation which projected into the lumen lined by atypical plumped endothelial cells. The vascular channels were also lined by plump cuboidal endothelial cells with focal hobnailed or “match-head” appearance. In some areas, endothelial cells formed solid-appearing aggregates with vessel lumens. By immunohistochemistry, the tumor cells were positive for CD31, CD34 and D2-40 at varying intensity. A final diagnosis of intraosseous PILA was made. To the best of our knowledge, this case is the first case of primary multifocal osseous PILA.

**Virtual slides:**

The virtual slide(s) for this article can be found here: http://www.diagnosticpathology.diagnomx.eu/vs/1919488629100787

## Background

Papillary intralymphatic angioendothelioma (PILA) or Dabska tumor is a locally aggressive, rarely metastasizing vascular lesion characterized by lymphatic- or vascular-like channels and papillary endothelial proliferation. The tumor is extremely rare, and often affects the skin and subcutaneous tissues of children [1]. Since its first description in 1969 by Dabska et al. only 33 cases have been described in the literature [2-13]. PILA does not appear to have any particular predilection site, but many of the reports were in dermis and subcutaneous tissues of head, neck, and extremities. Only a few cases of this tumor have also been described in deeper locations, including spleen, tongue, testis, and bone [8,10,11,13,18]. So far, only two cases of intraosseous PILA have been described in the literature, with none of these cases originating from facial bone. Herein, we present first case of multifocal PILA arising in facial bones of a 1-year old boy. The clinical and histological features of this tumor, as well as differential diagnosis are discussed.

## Case presentation

### Clinical presentation and management

**Figure 1:**
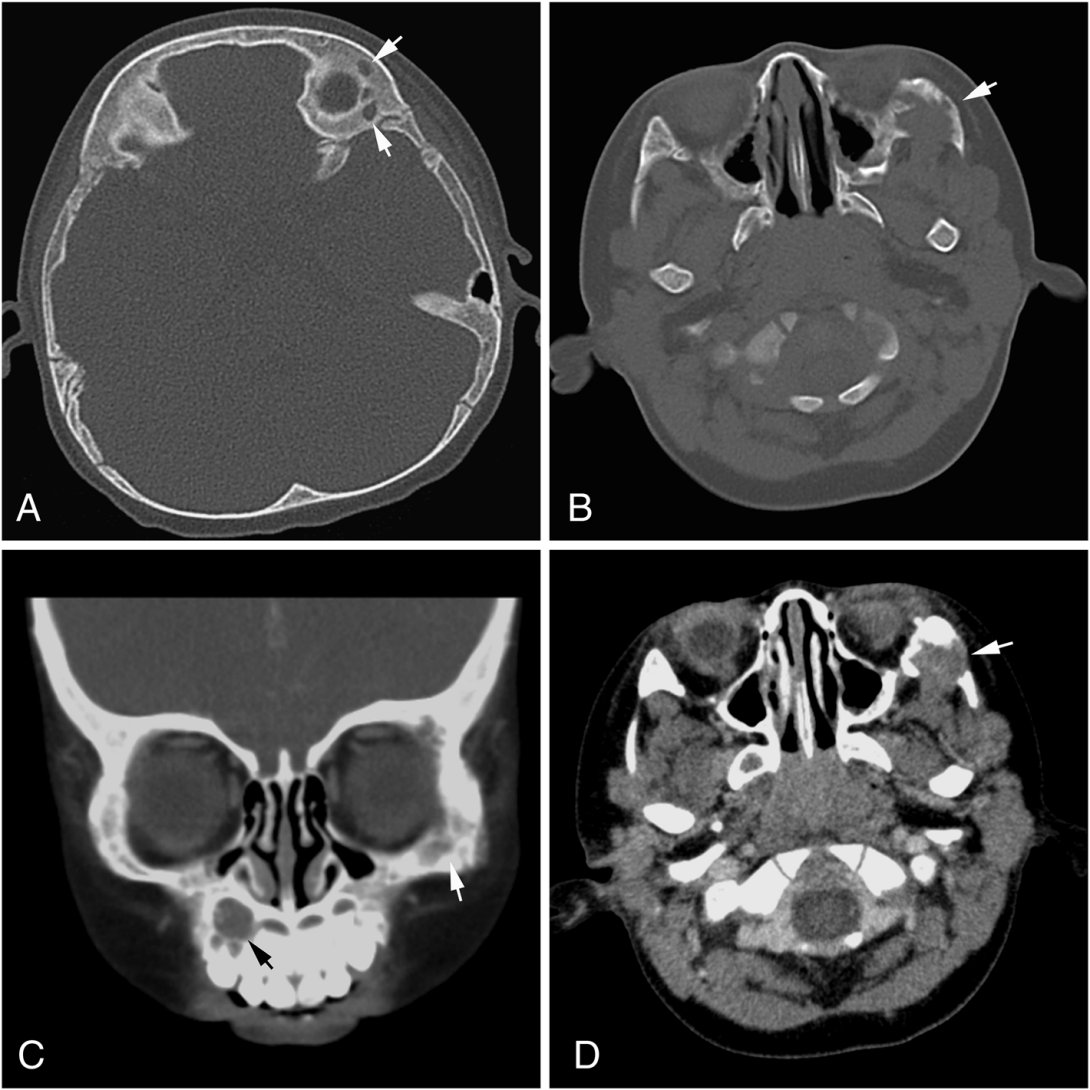
**Radiographic examination of the lesions. (A)** Cmputed tomography (CT) scan showed osteolytic lesions of the left orbital bone (white arrow). **(B)** A osteolytic lesion of the left zygomatic bone (white arrow) appeared to have an indistinct border in periphery. **(C)** Coronal CT scan showed multiple osseous destruction of maxillary bone (black arrow) and left zygomatic bone (white arrow). **(D)** Postcontrast axial CT scan in soft tissue windows revealed that an irregular mass destroyed the left zygomatic bone (white arrow).

A 1 year 3 month old Chinese male child was referred to our pediatric department for pain and swelling on his left side of face for past 2 weeks. In the past two weeks, the baby was suffering from a gradually severe soft tissues swelling on his left face. Two days before admission to our hospital, the pain and tenderness of left face became worse. As a result, the patient was referred to our hospital for examination and treatment. There was no history of any trauma to head and neck. Physical examination showed the patient had a mild soft tissues edema on his left upper face and severe pain was elicited upon pressure. There was no fever, weight loss and no palpable lymphadenopathy or organomegaly. The laboratory results, including blood count, differential, liver and renal function, were within the normal range. A computed tomography (CT) scan of the head revealed multifocal osteolytic lesions in the facial bones, including left zygomatic bone (measuring 1.5 × 1.0 × 1.0 cm in size), left orbital bone (measuring 0.5 cm in diameter) and right maxillary bone (measuring 1.0 cm in diameter). The most of left zygoma was observed to be destroyed and associated soft tissue mass was also noted (Figure 1). The lesions showed moderate enhancement after meglumine diatrizoate injection. There was no enlarged lymph node found in head and neck. A CT scan of neck and abdomen showed no pathologic findings, particularly no lymphadenopathy could be observed. A CT guided needle biopsy was performed on left zygomatic bone initially, but histopathological examination showed pieces of fibrosis with infiltration of inflammatory cells. From the clinical and radiographic evaluations, the lesion was preoperatively diagnosed as Langerhans cell histiocytosis (eosinophilic granuloma) of bone. The patient underwent curettage of the zygomatic and maxillary lesions. Because the margin of the lesions was ill-defined, the curettage was extensive. The postoperative phase was uneventful, and no additional treatments were undertaken. The pain resolved postoperatively and the patient was on regular follow-up for 24 months after discharging from hospital. A follow-up CT scan at 6 months after surgery revealed unchanged lesion of left orbital bone and there was no sign of recurrence of tumor and lymph node enlargement.

### Histopathological findings

The surgical samples were routinely fixed in 10% neutral buffered formalin. The tissues were embedded in paraffin. Four micrometer-thick sections were stained with H&E. Immunohistochemical analyses were performed using the ChemMate Envision/HRP Kit (Dako, Glostrup, Denmark). The antibodies used in this study included a broad panel of antibodies against cytokeratin (AE1/AE3), epithelial membrane antigen (EMA), vimentin, desmin, smooth muscle actin (SMA), CD31, CD34, D2-40, CD1a, CD68, S100 protein, Langerin, and Ki-67. The antibodies were obtained from Dako Cytomation (Glostrup, Denmark) and Santa Cruz Biotechnology (Santa Cruz, CA, USA).

**Figure 2:**
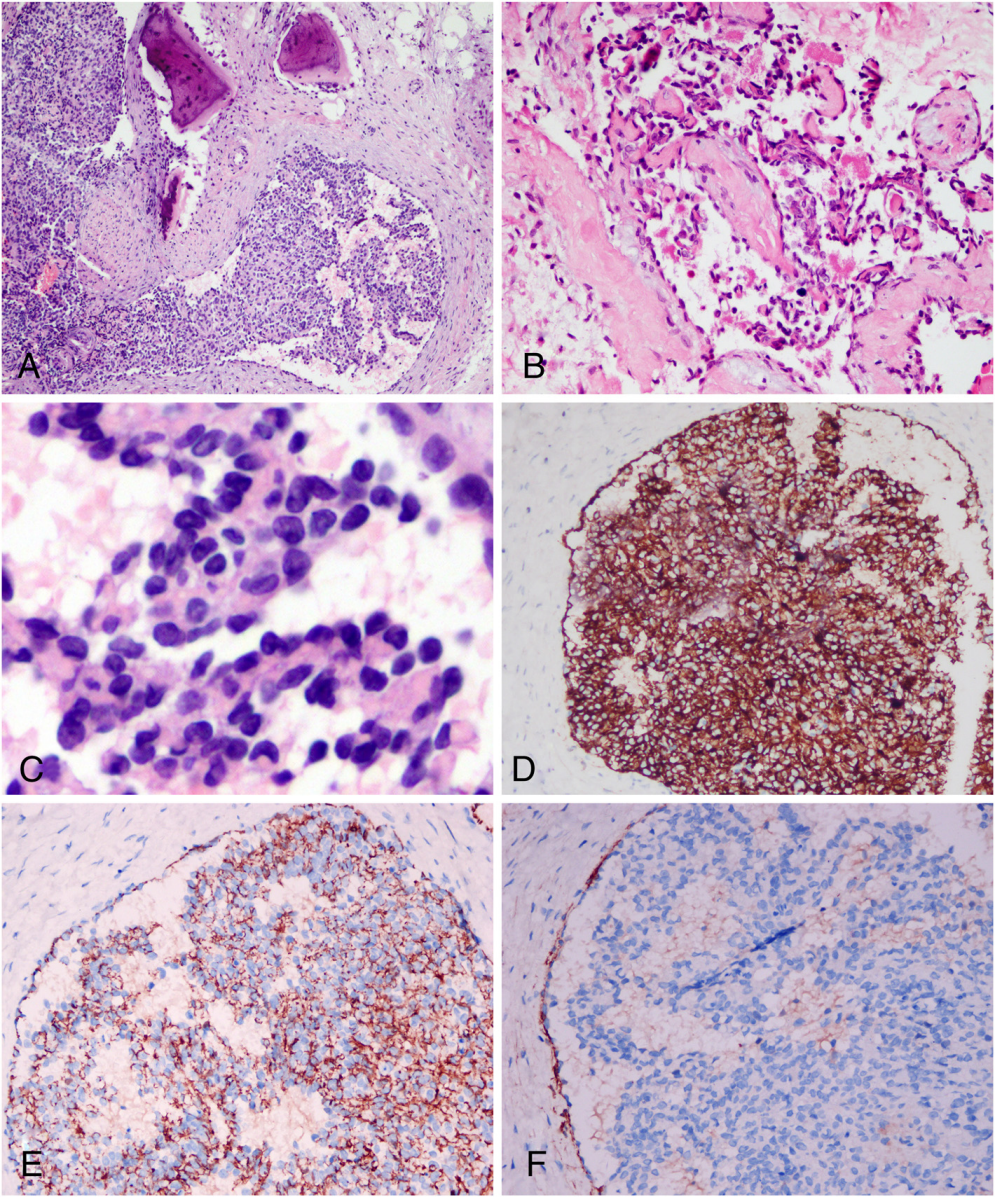
**Photomicrographs of the osseous lesions. (A)** Microscopic examination demonstrated ill-defined neoplasm composed of vascular lumen and endothelial cell proliferation forming glomerulus-like structures. **(B)** Delicate papillaries consisted of hyaline cores lined by prominent atypical nucleus and inconspicuous cytoplasm. **(C)** High-power photomicrograph demonstrated the characteristic “hobnail” appearance of the prominent endothelial cells. **(D)** Immunohistochemical staining showed the tumor cells strongly positive for CD31, but less positive for CD34 **(E)**. Endothelial cells of lumens expressed D2-40, but the intraluminal tumor cells were negative for D2-40. (**A**, HE staining with original magnification × 100; **B**, HE staining with original magnification × 200; **C**, HE staining with original magnification × 400; **D**-**F**, immunohistochemical staining with original magnification × 200).

Under microscopic examination, both zygomatic and maxillary lesion showed an ill-defined neoplasm within the osseous fragments. The lesions were composed of multiple delicate interconnecting vascular channels with papillae formation which projected into the lumen lined by atypical plumped endothelial cells. Some of those papillae contained hyalinized core. The vascular channels were also lined by plump cuboidal endothelial cells with focal hobnailed or “match-head” appearance. In some areas, endothelial cells formed solid-appearing aggregates with vessel lumens. A variable number of lymphocytes are seen within and around the vascular channels. Mitotic figures were rare and necrosis was not observed in the lesions (Figure 2A-C).

### Immunohistochemical findings

Immunohistochemistry stains done on these lesions were positivity for CD34 and CD31. Staining for CD31 was stronger than that for CD34. Endothelial cells of lumens expressed CD31, CD34 and D2-40, but the intraluminal tumor cells were negative for D2-40 (F). These lesions did not express CD1a, S-100 protein, cytokeratin (AE1/AE3), EMA, langerin, CD68 and SMA. The Ki-67 labeling index of the lesions was low, accounting for 2% of the tumor (Figure 2D-F). Based on the pathological findings, the mutifocal intraosseous lesions of facial bones were diagnosed as papillary intralymphatic angioendothelioma (PILA) according to WHO diagnostic criteria [1].

## Discussion

Papillary intralymphatic angioendothelioma (PILA), originally termed as endovascular papillary angioendothelioma (EPA), or Dabska tumor, was first presented in 1969 by Maria Dabska where she presented cases occurring in 6 children. In her originally reported cases, EPA was regarded as malignant and termed “malignant endovascular papillary angioendothelioma” because two of six patients had lymph node metastases [14]. Since then, a few reports of similar cases were published, often called “Dabska tumors.” In 1999, Fanburg-Smith JC and his colleagues described 12 similar cases and designated those tumors as “papillary intralymphatic angioendothelioma (PILA)” because of histologic and immunophenotypic evidence of lymphatic vessels [12]. Moreover, a growing number of cases described in the literature and the accumulating knowledge reveal that presence of highly specific lymphatic endothelial marker D2-40 and tumor marker vascular endothelial cell growth factor receptor type 3 (VEGFR 3) suggest tumor’s lymphatic origin other than hemangioma [15,16]. In 2002, World Health Organization (WHO) classification of tumors of soft tissue and bone accepted PILA as a rare entity, and identified it as a locally aggressive, rarely metastasizing “vascular lesion characterized by lymphatic-like channels and papillary endothelial proliferation” [17]. In 2013, the latest edition of WHO tumor classification (4^th^ edition) revised tumor description to “rarely metastasizing lymphatic vascular neoplasm”, which further supports lymphatic origin or differentiation of this tumor [1].

Since its first description by Dabska et al. only approximately 33, predominantly pediatric cases have been described in the literature. Of these 19 were children and 14 were adults without sex predilection. It appears that PILA may affect a wider age range than originally described by Dabska. The age range for all reported cases is from birth to 83 years. PILA does not show a preference for a specific anatomic site, but most of tumors involve the dermis and subcutaneous tissues of extremities (especially thigh and buttocks). The tumor has also been described in other deeper locations, including spleen [18], tongue [11], testis [8] and bone [10,13]. Intraosseous PILA confined to the bone is quite rare that only 2 cases have been reported worldwide to date. McCarthy et al. reported the first cases of Dabska tumor in the distal femur of a 45-year-old woman [13]. Also, Nakayama and his colleagues reported a lesion in the medial distal metaphysis of the femur in a 39-year-old woman [10]. In those cases, a single intraosseous lesion was observed in the involved bone (Table 1). However, there have been no reported cases of this lesion occurring in several facial bones with multiple foci. To the best of our knowledge, our case is the first case of primary multifocal osseous PILA. Clinically, PILA presents as a slowly growing asymptomatic cutaneous or soft tissue nodule, it usually comes to medical attention when significantly bigger in size (approximately 2–3 cm in diameter). Cases of reported PILA have ranged in size from 1 to 40 cm [12]. In the present case, pain and soft tissues swelling is the primary clinical manifestation instead of a palpable nodular lesion.

**Table 1:** Clinicopathological features of intraosseous PILAs described in present and previous reports

**No.**	**Authors (yr.)**	**Diagnosis**	**Age (year) /**	**Location**	**Clinical**	**Tumor**	**Immunophenotype**	**Treatment**	**Outcome**
			**Gender**		**presentation**	**size (cm)**			
1	McCarthy	EPA	45/Female	Left femoral	Left medial knee	1.5	CD31+	Complete	NED at 12
	EF (1999) [13]			condyle	pain for a year and			curettage	months
					tenderness over the				
					medial distal femur				
2	Nakayama T	EPA	39/Female	Right	Right knee	1.0	Vimentin+	Curettage	NED at 50
	(2004) [10]			epiphysis of	pain for a year		Factor VIII+	and re-excision	months
				femur				with a wider	
								margin	
3	The present case	PILA	1/Male	Left zygoma,	Pain and	0.5-1.5	Vimentin+,	Curettage	NED at 24
				left orbital	tenderness of		CD31+, CD34+,	with a	months
				bone and	left side of face		D2-40+	wider margin	
				right maxillary					

EPA, endovascular papillary angioendothelioma; PILA, papillary intralymphatic angioendothelioma; NED, no evidence of disease; +, positive.

Since cases of intraosseous PILA are so rare, the diagnosis should be only made by strict histological and clinical manifestation. According to the WHO criteria, PILA appears similar to cavernous lymphangiomas. The cuboidal or hobnail endothelial cells lining the vascular structures are characterized by a high nuclear cytoplasmic ratio and an apically placed nucleus that produces a surface bulge, accounting for the term "hobnail" or "matchstick". Immunohistochemically, vascular endothelial markers such as Von-Willebrand factor, CD34, CD31 and Fli-1 are commonly positive in most of tumors at varying intensities in each case, which help identify and diagnose the tumor as a type of vascular neoplasms. Of these markers, CD31 has been the best marker for this tumor because of its high sensitivity and specificity for vascular endothelial cells. CD31 usually has more immunohistochemical intensity than CD34 [12]. Moreover, the presence of podoplanin (D2-40) and vascular endothelial growth factor receptor-3 (VEGFR3) have been also confirmed by immunohis-tochemical staining, which are highly specific lymphatic endothelial markers and suggest that the tumor is more similar to a tumor of lymphatic origin [12,15]. However, the absence of D2-40 and CD34 in tumor cells could also be found in several previously reported cases [4]. In the present case, irregular vascular channels lined by endothelial cells with a “hobnail” appearance, papillary projections with a central hyaline core and scarce mitotic figures help identify the tumor as a “hobnail” hemangioendothelioma. The immuno-positivity of D2-40 points to a lymphatic origin of this tumor.

Intraosseous PILA usually show osteolysis in radiological examination (especially multifocal osteolytic lesions of bones in our current case), it is often misdiagnosed as eosinophilic granuloma of bone preoperatively. However, eosinophilic granuloma of bone shows clonal neoplastic proliferation of Langerhans cells that express CD1a, S-100 and Langerin. The absence of those specific Langerhans cell markers will rule out the diagnosis of eosinophilic granuloma. Importantly, two particular types of vascular lesions, retiform hemangioendothelioma and papillary endothelial hyperplasia (Masson's lesion), may also be confused with the PILA, because of presence of “hobnail” cells or irregular papillary projections in vascular channels. Retiform hemangioendothelioma is a locally aggressive vascular tumor and characterized by distinctive arborizing blood vessels lined by endothelial cell with “hobnail” morphology. The elongated, branched vessels impart a pattern reminiscent of rete testis. That histological appearance is not consistent to the PILA. Papillary endothelial hyperplasia, also known as Masson's lesion, is a reactive endothelial proliferation secondary to vascular thrombosis. The lesion shows branching papillary projections, many of which are free-floating in the vascular lumens. Thrombotic material is usually present in the vascular lumens. Moreover, these histologically similar vascular lesions do not express D2-40 and VEGFR3, which are specific markers for PILA. In addition, epithelioid hemangioendothelioma of bone has recently been reported in literature [19], and lymphangioma-like Kaposi sarcoma may infrequently be encountered in clinical practice [20]. These tumors might be confused with PILA because of endothelial cells proliferation and vascular structure with immunopositivity of vascular markers. However, presences of “hobnail” cells and papillary projections in vascular channels support the diagnosis of PILA.

Although PILA was identified initially as a “low-grade angiosarcoma”, following-up in more recent series demonstrated no local recurrence or metastasis [12]. Most of cases have excellent prognosis with complete, wide excision [13]. But high-grade angiosarcoma arising within a Dabska tumor suggests the tumor’s malignant potential in individual case [6]. Dabska performed a 30-year review of this tumor and the outcomes of the 6 patients she presented in 1969. Three of the original 6 cases were locally aggressive, with tumor invasion into deeper structures. Only one patient ultimately died of widespread pulmonary metastases [21]. However, Up to now, no other articles have been written describing the malignant metastatic potential of the tumor, although the tumor has been described to occur in lymph node [4]. In the present case there was no evidence of any nodal involvement or distant metastasis and the child was doing well on last follow up after 24 months of surgery. However, PILA can be locally invasive with a potential to metastasize, a long-term follow-up should be performed to supervise the locoregional recurrence.

## Conclusion

Because of its rarity and histological complexity, there exist diagnostic challenges for pathologists to differentiate PILA from other benign, intermediate or malignant vascular lesions. It is important to be able recognize this tumor in order to avoid potential misdiagnosis and improper management of afflicted patients. Our additive case is also presented for its rarity of site. It is the first case of intraosseous PILA with multiple lesions of facial bones. The diagnosis of intraosseous PILA is difficult and should be made cautiously. Besides confirmation by strict morphological criteria, immunohistochemical analysis is helpful for demonstrating the lymphatic phenotype of tumor.

### Consent

Written informed consent was obtained from the patient for publication of this case report and any accompanying images. A copy of the written consent is available for review by the Editor-in-Chief of this journal.

## Competing interests

The authors declare that we have no competing interests.

## Authors’ contributions

BL and YL made contributions to acquisition of clinical data, and analysis of the histological features by H&E staining and immunoassays. They are joint first co-authors and made an equal contribution to this work. XYT drafted the manuscript. ZL revised manuscript critically for important intellectual content and had given final approval of the version to be published. All authors read and approved the final manuscript.
